# PD03 Diagnostic Accuracy Of Liquid Biopsy In Breast, Lung, And Colorectal Cancer

**DOI:** 10.1017/S0266462325101414

**Published:** 2025-12-29

**Authors:** Anna Godo Pla, Maria Dolors Estrada Sabadell, Rosa Maria Vivanco-Hidalgo

## Abstract

**Introduction:**

Liquid biopsy (LB) is a minimally invasive technique that enables analysis of circulating tumor DNA (ctDNA) by next generation sequencing (NGS) and has several potential applications in oncology. The main objective was to describe the diagnostic accuracy of LB over tissue biopsy for screening, diagnosis, and tumor mutation profiling in breast, lung, and colorectal cancer, including at different cancer stages.

**Methods:**

Initially, a literature overview to select systematic reviews with meta-analyses or health technology assessment reports was conducted. When no data were obtained, a systematic review was conducted to select individual studies. For both procedures, a systematic literature search, two-step study selection, quality appraisal and a narrative synthesis were conducted.

**Results:**

The only study in the screening setting involved colorectal cancer (sensitivity 14%, specificity 90%). Regarding initial cancer diagnosis, LB in breast cancer had a sensitivity ranging from 31 to 74 percent and a specificity of 66 to 92 percent, with inconsistent results by stage. For lung cancer, LB had a sensitivity of 22 to 86 percent and a specificity of 42 to 91 percent, with improved utility in advanced stages. Colorectal cancer studies using multicomponent tests showed sensitivities of 83 to 93 percent and specificities of more than 95%. LB ctDNA analysis in molecular profiling of advanced cancer had high accuracy in breast (PIK3CA), lung (epidermal growth factor receptor mutations), and colorectal cancer (NRAS/KRAS), with values for sensitivity of up to 83 percent and for specificity of around 90 percent.

**Conclusions:**

No results were available for screening in breast and lung cancer. Limited evidence for colorectal cancer showed low sensitivity but high specificity for LB over tissue biopsy. Initial cancer diagnosis results were heterogeneous, with inconsistencies in breast and colorectal stages but improvements in advanced lung cancer. Tumor profiling provided the strongest evidence, with high diagnostic utility relative to tissue biopsy across all three cancer types.

